# 5-[(*E*)-2-Bromo­benzyl­idene]-8-(2-bromo­phen­yl)-2-hy­droxy-10-methyl-3,10-di­aza­hexa­cyclo­[10.7.1.1^3,7^.0^2,11^.0^7,11^.0^16,20^]henicosa-1(20),12,14,16,18-pentaen-6-one

**DOI:** 10.1107/S1600536810033295

**Published:** 2010-08-21

**Authors:** Raju Suresh Kumar, Hasnah Osman, Mohamed Ashraf Ali, Mohd Mustaqim Rosli, Hoong-Kun Fun

**Affiliations:** a School of Chemical Sciences, Universiti Sains Malaysia, 11800 USM, Penang, Malaysia; b Institute for Research in Molecular Medicine, Universiti Sains Malaysia, 11800 USM, Penang, Malaysia; c X-ray Crystallography Unit, School of Physics, Universiti Sains Malaysia, 11800 USM, Penang, Malaysia

## Abstract

In the title compound, C_33_H_26_Br_2_N_2_O_2_, the piperidine group adopts an envelope conformation while the two pyrrolidine groups adopt half-chair and envelope conformations. The dihydro­acenaphthyl­ene group is almost planar, with a maximum deviation of 0.105 (1) Å. The dihedral angle between the two bromo­phenyl rings is 60.19 (8)°. An intra­molecular O—H⋯N inter­action is observed, generating an *S*(5) ring motif. The crystal structure is stabilized by inter­molecular C—H⋯O inter­actions. Short Br⋯Br [3.461 (1) Å] and Br⋯C [3.322 (2) Å] inter­molecular contacts are observed, as well as π–π inter­actions [centroid–centroid distance = 3.793 (1) Å].

## Related literature

For biological studies of five-membered heterocycles, pyrrolidines and piperidines, see: Shi *et al.* (2009 ▶); Nair & Suja (2007 ▶); Nájera & Sansano (2005 ▶); Coldham & Hufton (2005 ▶); Daly *et al.* (1986 ▶); El-Subbagh *et al.* (2000 ▶); Dimmock *et al.* (2001 ▶). For ring puckering analysis, see: Cremer & Pople (1975 ▶). For the graph-set description of hydrogen-bond ring motifs, see: Bernstein *et al.* (1995 ▶). For a closely related crystal structure, see: Kumar *et al.* (2010 ▶). For the stability of the temperature controller used in the data collection, see: Cosier & Glazer (1986 ▶).
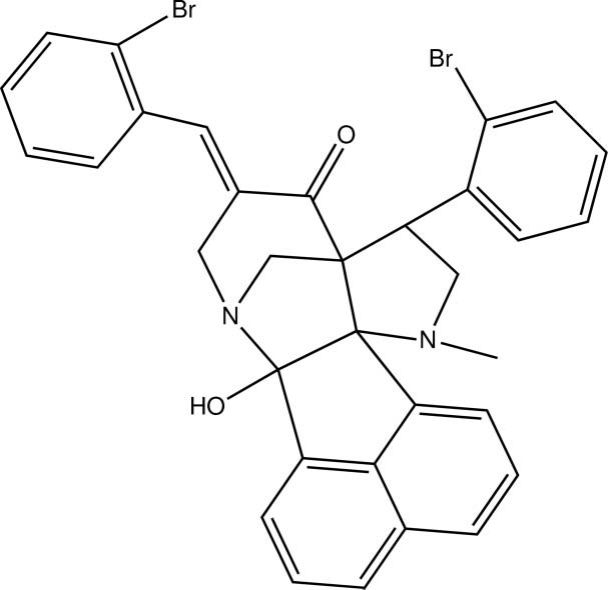


## Experimental

### 

#### Crystal data

C_33_H_26_Br_2_N_2_O_2_
*M* *_r_* = 642.38
Triclinic, 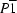

*a* = 8.3334 (14) Å
*b* = 12.4213 (19) Å
*c* = 12.8062 (19) Å
α = 80.623 (4)°
β = 79.787 (4)°
γ = 88.213 (4)°
*V* = 1287.2 (3) Å^3^
*Z* = 2
Mo *K*α radiation
μ = 3.19 mm^−1^
*T* = 100 K
0.52 × 0.41 × 0.19 mm

#### Data collection

Bruker APEXII DUO CCD area-detector diffractometer
Absorption correction: multi-scan (*SADABS*; Bruker, 2009 ▶) *T*_min_ = 0.288, *T*_max_ = 0.584
27238 measured reflections
9152 independent reflections
8233 reflections with *I* > 2σ(*I*)
*R*_int_ = 0.022

#### Refinement

*R*[*F* ^2^ > 2σ(*F* ^2^)] = 0.026
*wR*(*F* ^2^) = 0.105
*S* = 1.18
9152 reflections
357 parameters
H atoms treated by a mixture of independent and constrained refinement
Δρ_max_ = 0.99 e Å^−3^
Δρ_min_ = −1.17 e Å^−3^

### 

Data collection: *APEX2* (Bruker, 2009 ▶); cell refinement: *SAINT* (Bruker, 2009 ▶); data reduction: *SAINT*; program(s) used to solve structure: *SHELXTL* (Sheldrick, 2008 ▶); program(s) used to refine structure: *SHELXTL*; molecular graphics: *SHELXTL*; software used to prepare material for publication: *SHELXTL* and *PLATON* (Spek, 2009 ▶).

## Supplementary Material

Crystal structure: contains datablocks global, I. DOI: 10.1107/S1600536810033295/wn2405sup1.cif

Structure factors: contains datablocks I. DOI: 10.1107/S1600536810033295/wn2405Isup2.hkl

Additional supplementary materials:  crystallographic information; 3D view; checkCIF report

## Figures and Tables

**Table 1:** Hydrogen-bond geometry (Å, °)

*D*—H⋯*A*	*D*—H	H⋯*A*	*D*⋯*A*	*D*—H⋯*A*
O2—H1*O*2⋯N2	0.75 (3)	2.12 (3)	2.6735 (18)	132 (3)
C32—H32*A*⋯O2^i^	0.93	2.47	3.374 (2)	163
C33—H33*A*⋯O2^i^	0.96	2.55	3.292 (2)	134

Symmetry code: (i) 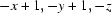.

**Fig. 1.**
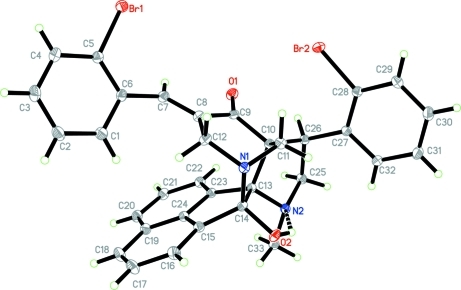
The molecular structure, showing 50% probability displacement ellipsoids and the atom-numbering scheme. Hydrogen atoms are shown as spheres of arbitrary radius.

**Fig. 2.**
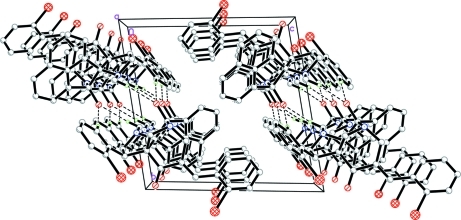
The crystal packing of the title compound, viewed down the a axis. Dashed lines indicate hydrogen bonds. H atoms not involved in the hydrogen bond interactions have been omitted for clarity.

## supplementary crystallographic information

### Comment

Multi-component 1,3-dipolar cycloaddition of ylidic species, such as azomethine ylides with olefinic dipolarophiles, plays a key role in the construction of biologically active five-membered heterocycles (Shi *et al.*, 2009; Nair *et al.*, 2007; Nájera *et al.*, 2005; Coldham *et al.*, 2005). Highly substituted pyrrolidines have gained much importance since they form the central skeleton of many natural products (Daly *et al.*, 1986). Heterocycles with the piperidine substructure display important biological activities, such as cytotoxic and anticancer activities (El-Subbagh *et al.*, 2000 ; Dimmock *et al.*, 2001). Because of the biological importance of these heterocycles, the crystal structure determination of the title compound was carried out and the results are presented in this paper.

All geometrical parameters of the title compound (Fig.1) are within normal ranges and comparable with those in a previously reported structure (Kumar *et al.*, 2010). The piperidine (N1/C8-C12) ring adopts an envelope confirmation [Q = 0.617 (2) Å, Θ = 44.6 (2)°, φ= 60.6 (2)°; Cremer & Pople, 1975]. The two five-membered pyrrolidine rings,  (N1/C10-C11/C13-C14) and (N2/C10/C13/C25-C26), adopt half-chair and envelope comformations respectively [Q = 0.452 (2) Å, φ= 198.8 (2)° and Q = 0.401 (2) Å, φ= 352.9 (2)°]. The dihydroacenaphthylene group, (C13-C24) is almost planar, with a maximum deviation of 0.105 (1) Å for atom C13. The dihedral angle between the two bromophenyl rings (C1-C6) and (C27-C32) is 60.19 (8)°.

An intramolecular O2—H1O2···N2 hydrogen bond (Table 1) forms a five-membered ring, generating an S(5) hydrogen bond ring motif (Bernstein *et al.*, 1995). In the crystal structure, molecules are connected by intermolecular C—H···O hydrogen bonds (Table 1, Fig. 2). Short intermolecular contacts Br2···Br2 [3.461 (1)Å] and Br1···C29 [3.322 (2)Å] are also observed. The crystal structure is further stabilized by π-π interactions with Cg1···Cg1 = 3.793 (1) Å, where Cg1 is the centroid of the C1-C6 benzene ring.

### Experimental

A mixture of 3,5-bis[(E)-(2-bromophenyl)methylidene]tetrahydro-4(1H)-pyridinone (0.100 g, 0.231 mmol), acenaphthenequinone (0.042 g, 0.231 mmol) and sarcosine (0.021 g, 0.231 mmol) was dissolved in methanol (10 ml) and refluxed for 1 hour. After completion of the reaction, as evident from TLC, the mixture was poured into water (50 ml). The precipitated solid was filtered, washed with water and recrystallised from a petroleum ether-ethyl acetate mixture (1:1) to yield the title compound as pale yellow crystals.

### Refinement

The H atom attached to O2 was located in a difference map and refined isotropically [O—H = 0.75 (3) Å]. The remaining H atoms were positioned geometrically and refined using a riding model [C—H = 0.93 Å for Csp^2^, 0.96 Å for methyl C, 0.97 Å for methylene C and 0.98 Å for methine C]; U_iso_(H) = xU_eq_(C), where x = 1.5 for methyl H and 1.2 for all other H atoms. A rotating model was used for the methyl group.

### Crystal data

**Table:**

C_33_H_26_Br_2_N_2_O_2_	*Z* = 2
*M**_r_* = 642.38	*F*(000) = 648
Triclinic, *P*1	*D*_x_ = 1.657 Mg m^−^^3^
Hall symbol: -P 1	Mo *K*α radiation, λ = 0.71073 Å
*a* = 8.3334 (14) Å	Cell parameters from 9995 reflections
*b* = 12.4213 (19) Å	θ = 3.3–35.1°
*c* = 12.8062 (19) Å	µ = 3.19 mm^−^^1^
α = 80.623 (4)°	*T* = 100 K
β = 79.787 (4)°	Block, yellow
γ = 88.213 (4)°	0.52 × 0.41 × 0.19 mm
*V* = 1287.2 (3) Å^3^	

### Data collection

**Table:**

Bruker APEXII DUO CCD area-detector diffractometer	9152 independent reflections
Radiation source: fine-focus sealed tube	8233 reflections with *I* > 2σ(*I*)
graphite	*R*_int_ = 0.022
φ and ω scans	θ_max_ = 32.5°, θ_min_ = 3.1°
Absorption correction: multi-scan (*SADABS*; Bruker, 2009)	*h* = −12→12
*T*_min_ = 0.288, *T*_max_ = 0.584	*k* = −18→18
27238 measured reflections	*l* = −19→19

### Refinement

**Table:**

Refinement on *F*^2^	Primary atom site location: structure-invariant direct methods
Least-squares matrix: full	Secondary atom site location: difference Fourier map
*R*[*F*^2^ > 2σ(*F*^2^)] = 0.026	Hydrogen site location: inferred from neighbouring sites
*wR*(*F*^2^) = 0.105	H atoms treated by a mixture of independent and constrained refinement
*S* = 1.18	*w* = 1/[σ^2^(*F*_o_^2^) + (0.0672*P*)^2^ + 0.2037*P*] where *P* = (*F*_o_^2^ + 2*F*_c_^2^)/3
9152 reflections	(Δ/σ)_max_ = 0.004
357 parameters	Δρ_max_ = 0.99 e Å^−^^3^
0 restraints	Δρ_min_ = −1.17 e Å^−^^3^

### Special details

**Table:**

Experimental. The crystal was placed in the cold stream of an Oxford Cyrosystems Cobra open-flow nitrogen cryostat (Cosier & Glazer, 1986) operating at 100.0 (1) K.
Geometry. All esds (except the esd in the dihedral angle between two l.s. planes) are estimated using the full covariance matrix. The cell esds are taken into account individually in the estimation of esds in distances, angles and torsion angles; correlations between esds in cell parameters are only used when they are defined by crystal symmetry. An approximate (isotropic) treatment of cell esds is used for estimating esds involving l.s. planes.
Refinement. Refinement of F^2^ against ALL reflections. The weighted R-factor wR and goodness of fit S are based on F^2^, conventional R-factors R are based on F, with F set to zero for negative F^2^. The threshold expression of F^2^ > 2sigma(F^2^) is used only for calculating R-factors(gt) etc. and is not relevant to the choice of reflections for refinement. R-factors based on F^2^ are statistically about twice as large as those based on F, and R- factors based on ALL data will be even larger.

### Fractional atomic coordinates and isotropic or equivalent isotropic displacement parameters (Å^2^)

**Table:**

	*x*	*y*	*z*	*U*_iso_*/*U*_eq_	
Br1	0.856585 (19)	1.068250 (12)	0.386199 (13)	0.01626 (5)	
Br2	1.025391 (18)	0.886819 (12)	−0.064263 (12)	0.01437 (5)	
O1	0.65922 (15)	0.94414 (9)	0.09019 (9)	0.0152 (2)	
O2	0.55092 (14)	0.49704 (9)	0.16200 (10)	0.0131 (2)	
N1	0.70944 (16)	0.61925 (10)	0.22074 (10)	0.0111 (2)	
N2	0.45256 (16)	0.66041 (10)	0.02417 (10)	0.0109 (2)	
C1	0.5433 (2)	0.80453 (13)	0.49862 (13)	0.0169 (3)	
H1A	0.4852	0.7568	0.4705	0.020*	
C2	0.5306 (2)	0.79520 (14)	0.60979 (14)	0.0193 (3)	
H2A	0.4666	0.7406	0.6551	0.023*	
C3	0.6137 (2)	0.86754 (14)	0.65250 (13)	0.0193 (3)	
H3A	0.6054	0.8614	0.7266	0.023*	
C4	0.7092 (2)	0.94912 (13)	0.58513 (13)	0.0173 (3)	
H4A	0.7636	0.9985	0.6138	0.021*	
C5	0.72276 (19)	0.95632 (12)	0.47468 (12)	0.0131 (2)	
C6	0.64227 (19)	0.88461 (12)	0.42811 (12)	0.0132 (2)	
C7	0.65161 (19)	0.89702 (12)	0.31078 (12)	0.0139 (3)	
H7A	0.6384	0.9672	0.2749	0.017*	
C8	0.67730 (18)	0.81722 (12)	0.25027 (12)	0.0124 (2)	
C9	0.67026 (18)	0.84990 (12)	0.13230 (12)	0.0117 (2)	
C10	0.66598 (17)	0.75367 (11)	0.07288 (11)	0.0104 (2)	
C11	0.78932 (18)	0.66652 (12)	0.11188 (12)	0.0122 (2)	
H11A	0.8086	0.6113	0.0655	0.015*	
H11B	0.8924	0.6998	0.1134	0.015*	
C12	0.71804 (19)	0.69952 (12)	0.29215 (12)	0.0135 (2)	
H12A	0.8275	0.6978	0.3084	0.016*	
H12B	0.6443	0.6764	0.3592	0.016*	
C13	0.49859 (17)	0.69498 (11)	0.11969 (11)	0.0101 (2)	
C14	0.54394 (17)	0.59505 (11)	0.20430 (11)	0.0106 (2)	
C15	0.41293 (18)	0.59321 (12)	0.30260 (12)	0.0115 (2)	
C16	0.3780 (2)	0.52059 (13)	0.39629 (13)	0.0161 (3)	
H16A	0.4395	0.4573	0.4076	0.019*	
C17	0.2453 (2)	0.54430 (14)	0.47598 (13)	0.0189 (3)	
H17A	0.2209	0.4953	0.5397	0.023*	
C18	0.1516 (2)	0.63738 (14)	0.46208 (13)	0.0182 (3)	
H18A	0.0664	0.6506	0.5161	0.022*	
C19	0.18568 (19)	0.71303 (12)	0.36501 (12)	0.0137 (2)	
C20	0.10036 (19)	0.81138 (13)	0.33826 (13)	0.0153 (3)	
H20A	0.0130	0.8321	0.3869	0.018*	
C21	0.14739 (19)	0.87631 (12)	0.23970 (13)	0.0148 (3)	
H21A	0.0902	0.9406	0.2237	0.018*	
C22	0.27944 (18)	0.84897 (12)	0.16176 (12)	0.0128 (2)	
H22A	0.3084	0.8945	0.0962	0.015*	
C23	0.36326 (17)	0.75363 (11)	0.18568 (11)	0.0106 (2)	
C24	0.31619 (18)	0.68768 (12)	0.28712 (12)	0.0113 (2)	
C25	0.50120 (18)	0.75089 (12)	−0.06406 (12)	0.0128 (2)	
H25A	0.4250	0.8112	−0.0589	0.015*	
H25B	0.5053	0.7274	−0.1331	0.015*	
C26	0.67185 (18)	0.78475 (12)	−0.05064 (11)	0.0112 (2)	
H26A	0.6810	0.8643	−0.0699	0.013*	
C27	0.81308 (18)	0.73321 (12)	−0.11802 (11)	0.0117 (2)	
C28	0.97428 (18)	0.76858 (12)	−0.12970 (12)	0.0120 (2)	
C29	1.10552 (19)	0.72043 (13)	−0.18784 (12)	0.0149 (3)	
H29A	1.2106	0.7463	−0.1930	0.018*	
C30	1.0790 (2)	0.63323 (13)	−0.23832 (13)	0.0173 (3)	
H30A	1.1661	0.5992	−0.2762	0.021*	
C31	0.9204 (2)	0.59773 (13)	−0.23128 (13)	0.0170 (3)	
H31A	0.9011	0.5409	−0.2666	0.020*	
C32	0.7901 (2)	0.64644 (13)	−0.17176 (12)	0.0150 (3)	
H32A	0.6851	0.6208	−0.1675	0.018*	
C33	0.28025 (18)	0.63124 (12)	0.03566 (12)	0.0132 (2)	
H33A	0.2641	0.6003	−0.0257	0.020*	
H33B	0.2139	0.6954	0.0405	0.020*	
H33C	0.2501	0.5789	0.0997	0.020*	
H1O2	0.547 (4)	0.516 (2)	0.104 (2)	0.028 (7)*	

### Atomic displacement parameters (Å^2^)

**Table:**

	*U*^11^	*U*^22^	*U*^33^	*U*^12^	*U*^13^	*U*^23^
Br1	0.01810 (8)	0.01305 (8)	0.01711 (8)	−0.00218 (5)	−0.00054 (6)	−0.00328 (6)
Br2	0.01454 (8)	0.01177 (8)	0.01788 (8)	−0.00154 (5)	−0.00447 (5)	−0.00345 (5)
O1	0.0200 (5)	0.0107 (4)	0.0152 (5)	−0.0001 (4)	−0.0042 (4)	−0.0015 (4)
O2	0.0173 (5)	0.0089 (4)	0.0143 (5)	0.0019 (4)	−0.0043 (4)	−0.0039 (4)
N1	0.0120 (5)	0.0103 (5)	0.0112 (5)	0.0008 (4)	−0.0027 (4)	−0.0017 (4)
N2	0.0113 (5)	0.0117 (5)	0.0100 (5)	−0.0005 (4)	−0.0021 (4)	−0.0017 (4)
C1	0.0182 (7)	0.0154 (6)	0.0167 (7)	−0.0008 (5)	−0.0006 (5)	−0.0035 (5)
C2	0.0225 (7)	0.0163 (7)	0.0164 (7)	−0.0009 (6)	0.0025 (6)	−0.0006 (5)
C3	0.0255 (8)	0.0194 (7)	0.0124 (6)	0.0030 (6)	−0.0032 (6)	−0.0016 (5)
C4	0.0206 (7)	0.0176 (7)	0.0148 (6)	0.0013 (5)	−0.0045 (5)	−0.0048 (5)
C5	0.0141 (6)	0.0113 (6)	0.0137 (6)	0.0014 (5)	−0.0021 (5)	−0.0024 (5)
C6	0.0149 (6)	0.0122 (6)	0.0126 (6)	0.0016 (5)	−0.0020 (5)	−0.0031 (5)
C7	0.0155 (6)	0.0122 (6)	0.0143 (6)	0.0001 (5)	−0.0031 (5)	−0.0027 (5)
C8	0.0138 (6)	0.0114 (6)	0.0125 (6)	−0.0003 (5)	−0.0035 (5)	−0.0021 (5)
C9	0.0107 (6)	0.0115 (6)	0.0135 (6)	−0.0013 (4)	−0.0026 (5)	−0.0031 (5)
C10	0.0110 (5)	0.0094 (5)	0.0107 (6)	0.0002 (4)	−0.0019 (4)	−0.0016 (4)
C11	0.0111 (6)	0.0120 (6)	0.0131 (6)	0.0015 (5)	−0.0028 (5)	−0.0008 (5)
C12	0.0167 (6)	0.0115 (6)	0.0135 (6)	−0.0003 (5)	−0.0058 (5)	−0.0026 (5)
C13	0.0112 (5)	0.0083 (5)	0.0105 (5)	0.0008 (4)	−0.0014 (4)	−0.0011 (4)
C14	0.0118 (6)	0.0081 (5)	0.0124 (6)	0.0010 (4)	−0.0029 (4)	−0.0024 (4)
C15	0.0122 (6)	0.0103 (5)	0.0118 (6)	0.0002 (4)	−0.0018 (5)	−0.0011 (4)
C16	0.0190 (7)	0.0131 (6)	0.0151 (6)	0.0017 (5)	−0.0027 (5)	0.0004 (5)
C17	0.0198 (7)	0.0188 (7)	0.0146 (7)	0.0006 (6)	0.0007 (5)	0.0031 (5)
C18	0.0180 (7)	0.0197 (7)	0.0142 (6)	0.0014 (6)	0.0019 (5)	−0.0002 (5)
C19	0.0133 (6)	0.0135 (6)	0.0141 (6)	0.0007 (5)	−0.0009 (5)	−0.0031 (5)
C20	0.0139 (6)	0.0151 (6)	0.0175 (7)	0.0030 (5)	−0.0019 (5)	−0.0059 (5)
C21	0.0145 (6)	0.0130 (6)	0.0178 (7)	0.0042 (5)	−0.0039 (5)	−0.0045 (5)
C22	0.0142 (6)	0.0108 (6)	0.0137 (6)	0.0017 (5)	−0.0037 (5)	−0.0015 (5)
C23	0.0109 (5)	0.0095 (5)	0.0116 (6)	0.0008 (4)	−0.0022 (4)	−0.0024 (4)
C24	0.0115 (6)	0.0105 (5)	0.0121 (6)	0.0005 (4)	−0.0025 (5)	−0.0017 (5)
C25	0.0126 (6)	0.0140 (6)	0.0112 (6)	−0.0001 (5)	−0.0024 (5)	−0.0002 (5)
C26	0.0119 (6)	0.0109 (5)	0.0104 (6)	0.0003 (4)	−0.0017 (4)	−0.0012 (4)
C27	0.0132 (6)	0.0110 (6)	0.0104 (6)	−0.0011 (5)	−0.0011 (5)	−0.0010 (4)
C28	0.0136 (6)	0.0104 (6)	0.0117 (6)	0.0001 (5)	−0.0022 (5)	−0.0013 (5)
C29	0.0132 (6)	0.0157 (6)	0.0147 (6)	0.0007 (5)	−0.0006 (5)	−0.0014 (5)
C30	0.0191 (7)	0.0164 (7)	0.0158 (7)	0.0023 (5)	0.0000 (5)	−0.0041 (5)
C31	0.0211 (7)	0.0141 (6)	0.0159 (6)	−0.0006 (5)	−0.0009 (5)	−0.0053 (5)
C32	0.0160 (6)	0.0143 (6)	0.0147 (6)	−0.0029 (5)	−0.0004 (5)	−0.0046 (5)
C33	0.0113 (6)	0.0137 (6)	0.0149 (6)	−0.0007 (5)	−0.0022 (5)	−0.0037 (5)

### Geometric parameters (Å, °)

**Table:**

Br1—C5	1.8963 (15)	C14—C15	1.511 (2)
Br2—C28	1.8993 (15)	C15—C16	1.370 (2)
O1—C9	1.2144 (18)	C15—C24	1.410 (2)
O2—C14	1.4065 (17)	C16—C17	1.424 (2)
O2—H1O2	0.75 (3)	C16—H16A	0.9300
N1—C11	1.4697 (19)	C17—C18	1.380 (2)
N1—C12	1.4698 (19)	C17—H17A	0.9300
N1—C14	1.4785 (19)	C18—C19	1.421 (2)
N2—C33	1.4680 (19)	C18—H18A	0.9300
N2—C25	1.4684 (19)	C19—C24	1.405 (2)
N2—C13	1.4750 (19)	C19—C20	1.418 (2)
C1—C2	1.395 (2)	C20—C21	1.382 (2)
C1—C6	1.407 (2)	C20—H20A	0.9300
C1—H1A	0.9300	C21—C22	1.421 (2)
C2—C3	1.385 (3)	C21—H21A	0.9300
C2—H2A	0.9300	C22—C23	1.3749 (19)
C3—C4	1.388 (2)	C22—H22A	0.9300
C3—H3A	0.9300	C23—C24	1.416 (2)
C4—C5	1.387 (2)	C25—C26	1.542 (2)
C4—H4A	0.9300	C25—H25A	0.9700
C5—C6	1.397 (2)	C25—H25B	0.9700
C6—C7	1.474 (2)	C26—C27	1.521 (2)
C7—C8	1.344 (2)	C26—H26A	0.9800
C7—H7A	0.9300	C27—C28	1.402 (2)
C8—C9	1.510 (2)	C27—C32	1.405 (2)
C8—C12	1.523 (2)	C28—C29	1.387 (2)
C9—C10	1.522 (2)	C29—C30	1.391 (2)
C10—C11	1.554 (2)	C29—H29A	0.9300
C10—C26	1.558 (2)	C30—C31	1.389 (2)
C10—C13	1.569 (2)	C30—H30A	0.9300
C11—H11A	0.9700	C31—C32	1.394 (2)
C11—H11B	0.9700	C31—H31A	0.9300
C12—H12A	0.9700	C32—H32A	0.9300
C12—H12B	0.9700	C33—H33A	0.9600
C13—C23	1.526 (2)	C33—H33B	0.9600
C13—C14	1.592 (2)	C33—H33C	0.9600
			
C14—O2—H1O2	103 (2)	C16—C15—C14	132.30 (13)
C11—N1—C12	108.02 (12)	C24—C15—C14	108.17 (12)
C11—N1—C14	102.44 (11)	C15—C16—C17	118.42 (14)
C12—N1—C14	115.63 (11)	C15—C16—H16A	120.8
C33—N2—C25	112.31 (12)	C17—C16—H16A	120.8
C33—N2—C13	114.95 (12)	C18—C17—C16	122.38 (15)
C25—N2—C13	105.24 (11)	C18—C17—H17A	118.8
C2—C1—C6	121.38 (16)	C16—C17—H17A	118.8
C2—C1—H1A	119.3	C17—C18—C19	119.82 (14)
C6—C1—H1A	119.3	C17—C18—H18A	120.1
C3—C2—C1	119.73 (16)	C19—C18—H18A	120.1
C3—C2—H2A	120.1	C24—C19—C20	116.51 (14)
C1—C2—H2A	120.1	C24—C19—C18	116.89 (14)
C2—C3—C4	120.28 (15)	C20—C19—C18	126.60 (14)
C2—C3—H3A	119.9	C21—C20—C19	119.84 (14)
C4—C3—H3A	119.9	C21—C20—H20A	120.1
C5—C4—C3	119.32 (15)	C19—C20—H20A	120.1
C5—C4—H4A	120.3	C20—C21—C22	122.79 (14)
C3—C4—H4A	120.3	C20—C21—H21A	118.6
C4—C5—C6	122.33 (14)	C22—C21—H21A	118.6
C4—C5—Br1	117.66 (12)	C23—C22—C21	118.38 (14)
C6—C5—Br1	120.00 (11)	C23—C22—H22A	120.8
C5—C6—C1	116.92 (14)	C21—C22—H22A	120.8
C5—C6—C7	121.47 (14)	C22—C23—C24	118.82 (13)
C1—C6—C7	121.48 (14)	C22—C23—C13	132.51 (13)
C8—C7—C6	126.64 (14)	C24—C23—C13	108.52 (12)
C8—C7—H7A	116.7	C19—C24—C15	122.94 (13)
C6—C7—H7A	116.7	C19—C24—C23	123.65 (13)
C7—C8—C9	116.44 (13)	C15—C24—C23	113.41 (13)
C7—C8—C12	124.44 (14)	N2—C25—C26	105.15 (11)
C9—C8—C12	119.08 (12)	N2—C25—H25A	110.7
O1—C9—C8	122.98 (13)	C26—C25—H25A	110.7
O1—C9—C10	123.03 (13)	N2—C25—H25B	110.7
C8—C9—C10	113.85 (12)	C26—C25—H25B	110.7
C9—C10—C11	108.11 (12)	H25A—C25—H25B	108.8
C9—C10—C26	114.98 (11)	C27—C26—C25	114.83 (12)
C11—C10—C26	117.57 (11)	C27—C26—C10	113.94 (12)
C9—C10—C13	106.44 (11)	C25—C26—C10	102.43 (11)
C11—C10—C13	101.86 (11)	C27—C26—H26A	108.4
C26—C10—C13	106.49 (11)	C25—C26—H26A	108.4
N1—C11—C10	104.01 (11)	C10—C26—H26A	108.4
N1—C11—H11A	111.0	C28—C27—C32	115.83 (13)
C10—C11—H11A	111.0	C28—C27—C26	122.18 (13)
N1—C11—H11B	111.0	C32—C27—C26	121.98 (13)
C10—C11—H11B	111.0	C29—C28—C27	123.13 (14)
H11A—C11—H11B	109.0	C29—C28—Br2	115.84 (11)
N1—C12—C8	116.67 (12)	C27—C28—Br2	121.03 (11)
N1—C12—H12A	108.1	C28—C29—C30	119.60 (15)
C8—C12—H12A	108.1	C28—C29—H29A	120.2
N1—C12—H12B	108.1	C30—C29—H29A	120.2
C8—C12—H12B	108.1	C31—C30—C29	119.04 (15)
H12A—C12—H12B	107.3	C31—C30—H30A	120.5
N2—C13—C23	114.26 (12)	C29—C30—H30A	120.5
N2—C13—C10	102.66 (11)	C30—C31—C32	120.60 (15)
C23—C13—C10	119.66 (11)	C30—C31—H31A	119.7
N2—C13—C14	112.84 (11)	C32—C31—H31A	119.7
C23—C13—C14	103.45 (11)	C31—C32—C27	121.75 (15)
C10—C13—C14	103.78 (11)	C31—C32—H32A	119.1
O2—C14—N1	108.22 (11)	C27—C32—H32A	119.1
O2—C14—C15	112.29 (12)	N2—C33—H33A	109.5
N1—C14—C15	114.63 (12)	N2—C33—H33B	109.5
O2—C14—C13	110.91 (11)	H33A—C33—H33B	109.5
N1—C14—C13	105.17 (11)	N2—C33—H33C	109.5
C15—C14—C13	105.37 (11)	H33A—C33—H33C	109.5
C16—C15—C24	119.53 (14)	H33B—C33—H33C	109.5
			
C6—C1—C2—C3	1.5 (3)	C10—C13—C14—C15	135.81 (11)
C1—C2—C3—C4	0.0 (3)	O2—C14—C15—C16	51.9 (2)
C2—C3—C4—C5	−1.0 (3)	N1—C14—C15—C16	−72.2 (2)
C3—C4—C5—C6	0.5 (2)	C13—C14—C15—C16	172.71 (16)
C3—C4—C5—Br1	−179.35 (13)	O2—C14—C15—C24	−128.68 (13)
C4—C5—C6—C1	1.0 (2)	N1—C14—C15—C24	107.29 (14)
Br1—C5—C6—C1	−179.16 (11)	C13—C14—C15—C24	−7.84 (15)
C4—C5—C6—C7	176.88 (15)	C24—C15—C16—C17	−0.9 (2)
Br1—C5—C6—C7	−3.2 (2)	C14—C15—C16—C17	178.52 (16)
C2—C1—C6—C5	−2.0 (2)	C15—C16—C17—C18	−0.1 (3)
C2—C1—C6—C7	−177.90 (15)	C16—C17—C18—C19	0.5 (3)
C5—C6—C7—C8	135.05 (17)	C17—C18—C19—C24	0.0 (2)
C1—C6—C7—C8	−49.2 (2)	C17—C18—C19—C20	179.44 (16)
C6—C7—C8—C9	175.99 (14)	C24—C19—C20—C21	0.0 (2)
C6—C7—C8—C12	−6.2 (3)	C18—C19—C20—C21	−179.40 (16)
C7—C8—C9—O1	7.5 (2)	C19—C20—C21—C22	0.2 (2)
C12—C8—C9—O1	−170.38 (14)	C20—C21—C22—C23	0.2 (2)
C7—C8—C9—C10	−168.33 (13)	C21—C22—C23—C24	−0.9 (2)
C12—C8—C9—C10	13.77 (19)	C21—C22—C23—C13	174.12 (15)
O1—C9—C10—C11	142.12 (15)	N2—C13—C23—C22	−61.5 (2)
C8—C9—C10—C11	−42.03 (16)	C10—C13—C23—C22	60.7 (2)
O1—C9—C10—C26	8.5 (2)	C14—C13—C23—C22	175.42 (15)
C8—C9—C10—C26	−175.62 (12)	N2—C13—C23—C24	113.85 (13)
O1—C9—C10—C13	−109.10 (16)	C10—C13—C23—C24	−123.91 (13)
C8—C9—C10—C13	66.74 (15)	C14—C13—C23—C24	−9.21 (15)
C12—N1—C11—C10	−73.52 (14)	C20—C19—C24—C15	179.51 (14)
C14—N1—C11—C10	49.00 (13)	C18—C19—C24—C15	−1.0 (2)
C9—C10—C11—N1	73.21 (14)	C20—C19—C24—C23	−0.8 (2)
C26—C10—C11—N1	−154.57 (12)	C18—C19—C24—C23	178.75 (15)
C13—C10—C11—N1	−38.68 (13)	C16—C15—C24—C19	1.5 (2)
C11—N1—C12—C8	44.67 (17)	C14—C15—C24—C19	−178.07 (14)
C14—N1—C12—C8	−69.37 (17)	C16—C15—C24—C23	−178.31 (14)
C7—C8—C12—N1	168.00 (15)	C14—C15—C24—C23	2.16 (17)
C9—C8—C12—N1	−14.3 (2)	C22—C23—C24—C19	1.2 (2)
C33—N2—C13—C23	−31.95 (17)	C13—C23—C24—C19	−174.92 (14)
C25—N2—C13—C23	92.16 (14)	C22—C23—C24—C15	−179.05 (14)
C33—N2—C13—C10	−163.06 (11)	C13—C23—C24—C15	4.84 (17)
C25—N2—C13—C10	−38.96 (13)	C33—N2—C25—C26	169.39 (11)
C33—N2—C13—C14	85.87 (14)	C13—N2—C25—C26	43.64 (14)
C25—N2—C13—C14	−150.02 (12)	N2—C25—C26—C27	95.36 (14)
C9—C10—C13—N2	143.27 (11)	N2—C25—C26—C10	−28.69 (13)
C11—C10—C13—N2	−103.58 (12)	C9—C10—C26—C27	122.63 (13)
C26—C10—C13—N2	20.15 (13)	C11—C10—C26—C27	−6.42 (18)
C9—C10—C13—C23	15.49 (17)	C13—C10—C26—C27	−119.76 (13)
C11—C10—C13—C23	128.64 (13)	C9—C10—C26—C25	−112.74 (13)
C26—C10—C13—C23	−107.63 (14)	C11—C10—C26—C25	118.21 (13)
C9—C10—C13—C14	−99.04 (12)	C13—C10—C26—C25	4.87 (13)
C11—C10—C13—C14	14.11 (13)	C25—C26—C27—C28	168.94 (13)
C26—C10—C13—C14	137.84 (11)	C10—C26—C27—C28	−73.35 (17)
C11—N1—C14—O2	79.77 (13)	C25—C26—C27—C32	−11.6 (2)
C12—N1—C14—O2	−163.02 (12)	C10—C26—C27—C32	106.11 (16)
C11—N1—C14—C15	−154.06 (12)	C32—C27—C28—C29	−1.7 (2)
C12—N1—C14—C15	−36.85 (17)	C26—C27—C28—C29	177.82 (14)
C11—N1—C14—C13	−38.81 (13)	C32—C27—C28—Br2	178.85 (11)
C12—N1—C14—C13	78.40 (14)	C26—C27—C28—Br2	−1.7 (2)
N2—C13—C14—O2	7.93 (16)	C27—C28—C29—C30	0.5 (2)
C23—C13—C14—O2	131.93 (12)	Br2—C28—C29—C30	179.96 (12)
C10—C13—C14—O2	−102.44 (13)	C28—C29—C30—C31	1.4 (2)
N2—C13—C14—N1	124.69 (12)	C29—C30—C31—C32	−2.0 (3)
C23—C13—C14—N1	−111.31 (12)	C30—C31—C32—C27	0.7 (3)
C10—C13—C14—N1	14.32 (13)	C28—C27—C32—C31	1.1 (2)
N2—C13—C14—C15	−113.81 (13)	C26—C27—C32—C31	−178.41 (15)
C23—C13—C14—C15	10.19 (14)		

### Hydrogen-bond geometry (Å, °)

**Table:**

*D*—H···*A*	*D*—H	H···*A*	*D*···*A*	*D*—H···*A*
O2—H1O2···N2	0.75 (3)	2.12 (3)	2.6735 (18)	132 (3)
C32—H32A···O2^i^	0.93	2.47	3.374 (2)	163
C33—H33A···O2^i^	0.96	2.55	3.292 (2)	134

Symmetry codes: (i) −*x*+1, −*y*+1, −*z*.
